# Higher mucosal antibody concentrations in women with genital tract inflammation

**DOI:** 10.1038/s41598-021-02954-0

**Published:** 2021-12-06

**Authors:** Parveen Sobia, Thevani Pillay, Lenine J. P. Liebenberg, Aida Sivro, Leila E. Mansoor, Farzana Osman, Jo-Ann S. Passmore, Quarraisha Abdool Karim, Salim S. Abdool Karim, Cheryl Baxter, Lyle R. McKinnon, Derseree Archary

**Affiliations:** 1 Centre for the AIDS Programme of Research in South Africa (CAPRISA), University of KwaZulu-Natal, 2nd Floor, Doris Duke Medical Research Institute, 719 Umbilo Road, Durban, 4041 South Africa; 2 Department of Medical Microbiology, University of Kwazulu-Natal, Durban, South Africa; 3 Department of Medical Microbiology and Infectious Diseases, University of Manitoba, Winnipeg, MB Canada; 4 Institute of Infectious Diseases and Molecular Medicine (IDM), University of Cape Town, Cape Town, South Africa; 5 Department of Epidemiology, Columbia University, New York, NY USA; 6 National HIV and Retrovirology Labs, JC Wilt Infectious Disease Research Centre, Public Health Agency of Canada, Winnipeg, MB Canada

**Keywords:** Immunology, Diseases

## Abstract

Inflammatory cytokines augment humoral responses by stimulating antibody production and inducing class-switching. In women, genital inflammation (GI) significantly modifies HIV risk. However, the impact of GI on mucosal antibodies remains undefined. We investigated the impact of GI, pre-HIV infection, on antibody isotypes and IgG subclasses in the female genital tract. Immunoglobulin (Ig) isotypes, IgG subclasses and 48 cytokines were measured prior to HIV infection in cervicovaginal lavages (CVL) from 66 HIV seroconverters (cases) and 66 matched HIV-uninfected women (controls) enrolled in the CAPRISA 004 and 008 1% tenofovir gel trials. Pre-HIV infection, cases had significantly higher genital IgM (4.13; IQR, 4.04–4.19) compared to controls (4.06; IQR, 3.90–4.20; *p* = 0.042). More than one-quarter of cases (27%) had GI compared to just over one-tenth (12%) in controls. Significantly higher IgG1, IgG3, IgG4 and IgM (all *p* < 0.05) were found in women stratified for GI compared to women without. Adjusted linear mixed models showed several pro-inflammatory, chemotactic, growth factors, and adaptive cytokines significantly correlated with higher titers of IgM, IgA and IgG subclasses (*p* < 0.05). The strong and significant positive correlations between mucosal antibodies and markers of GI suggest that GI may impact mucosal antibody profiles. These findings require further investigation to establish a plausible biological link between the local inflammatory milieu and its consequence on these genital antibodies.

## Introduction

Women and girls account for approximately half of all new HIV infections globally. In sub-Saharan Africa, young women aged 15–24 years are twice as likely to be HIV infected than similarly aged men and account for 24% of all new HIV infections^1^. Understanding the biology underlying the vulnerability of the female genital tract (FGT), the predominant site of heterosexual HIV transmission, is thus a major focus for HIV prevention efforts.

Genital inflammation (GI) defined as elevated levels of five of nine key pro-inflammatory cytokines in the FGT confers a three-fold higher-risk of HIV acquisition^2^. While the causes of GI remain mostly undefined, attributable factors for pre-existing GI include sexually transmitted infections (STIs)^3^, vaginal microbial dysbiosis^3^, hormonal contraceptive use^4^ and various sexual practices^5^. GI was previously established as a significant predictor of HIV acquisition in women from the CAP004 tenofovir gel trial^2^. Further cohort analyses showed that women without GI had 75% protection from HIV infection compared to − 10% in women with GI, despite a similarly high tenofovir gel adherence^6^. In HIV-uninfected women, elevated genital cytokines were significantly associated with increased mucosal target CD4+T cells and markers for epithelial barrier disruption^7^ supporting the plausible link between GI and enhanced HIV risk.

Antibodies in the FGT may play an important role in protection against mucosal transmission of HIV^8^. In addition to viral neutralization, antibodies can confer non-neutralizing effector functions such as antibody-dependent cellular phagocytosis (ADCP) and antibody dependent cellular cytotoxicity (ADCC)^9,10^. Among the immunoglobulins, IgM is the first to appear in response to any infection, followed by cytokine-mediated class switching to IgG and IgA^11–13^. Despite relatively high IgA, IgG predominates in the FGT^14,15^. Several studies have attributed the presence of genital tract HIV-specific IgA as a correlate of protection in highly exposed persistently seronegative (HEPS) women^16–18^. In the RV144 trial vaccinees, circulating Env V1-V2 IgG correlated with lower HIV-1 risk through enhanced ADCC, ADCP and complement activation^19,20^. Whether these antibodies transduced from the circulation to the mucosal compartment to confer protection remains undefined. Collectively these findings underscore the importance of both locally and/or transduced antibody responses which may help to mitigate HIV infections.

Inflammatory cytokines and chemokines influence B cell functions, including antibody production, isotype and IgG subclass switching^21,22^. Previously, cytokines were shown to influence effector functions of antibodies in the HIV-infected individuals^23^. Elevated inflammatory cytokines in the FGT were also associated with increased levels of protease expression leading to loss of antibody functions through proteolytic cleavage^7,24^. Our group showed significant associations between antibodies and pro-inflammatory cytokines in the semen of HIV-infected men^25^ shedding light on the profile of mucosal antibodies in relation to local cytokines. Less well defined is the relationship between local cytokines and humoral immunity in the FGT. In this study we measured the antibody isotype and subclasses in the FGT of women from the CAPRISA 004 and CAPRISA 008 1% tenofovir gel trials^26,27^ in relation to the mucosal cytokines that are used to gauge GI. Understanding the relationship between pre-existing GI and antibody isotypes and subclasses in the FGT are also important to the design of more effective prevention strategies.

## Methods

### Study participants and sample collection

This is a retrospective sub-study of N = 132 women from the CAPRISA 004 and CAPRISA 008 clinical trial studies. CAPRISA 004 was a randomized, double-blinded, placebo-controlled trial assessing the effectiveness of tenofovir gel to prevent HIV^27^. CAPRISA 008 was a two-arm, open-label, randomized controlled, non-inferiority implementation trial assessing whether tenofovir gel provision could be effectively integrated into family planning clinics^26^. All participants provided written informed consent for study participation and the sub-study was approved by the Biomedical Research Ethics Committee of University of KwaZulu-Natal (BE0207/17). All the experimental procedures are in accordance with the relevant ethical guidelines and regulations.

Cervico-vaginal lavage (CVL) samples were obtained at the pre-infection timepoint from women who subsequently became HIV-infected (cases, n = 66) and women who remained uninfected (controls, n = 66). Pre-infection CVLs for each of the cases were the last HIV negative samples before testing HIV positive by PCR. The cases and controls were matched by time in study and tenofovir use. In controls, their timepoint was matched to the pre-infection timepoint for the cases. All the samples were chosen according to a 1:1 ratio of cases to controls. CVL samples were collected and stored from each woman according to the method described by Bebell et al^28^, CVL sample collection was deferred if women were menstruating.

### IgG subclasses and Ig isotype quantification in CVL

IgG subclasses (IgG1, IgG2, IgG3 and IgG4) and isotypes IgA and IgM in CVL were quantified using the Bio-Plex Pro™ Human Isotyping Panel kit (Bio-Rad, USA) according to the manufacturer’s instructions. The Bio-Plex 200 multiplex system (Bio-Rad, Hercules, CA) was used to determine the levels of immunoglobulins. Mean Fluorescent Intensity (MFI) were determined by 4-PL logistic regression using the Bioplex Manager 6.0 software (Bio-Rad, Hercules, CA). CVL samples were diluted at 1:10 in sterile PBS to ensure that the MFI for the IgG subclasses and isotypes were detected in the linear range of the standard curve.

### Genital cytokine measurement by multiplex ELISA

Concentrations of 48 cytokines involved in different immunological functions were measured using Bio-Plex Pro™ Human Cytokine Group I 27-Plex Panel and Group II 21-Plex Panel (Bio-Rad, USA) and Single-plex Human Magnetic Luminex Assay (R&D systems, USA) in the CVLs. The cytokine panel included: interleukin (IL)-1β, IL-1Rα, IL-2, IL-4, IL-5, IL-6, IL-7, IL-8, IL-9, IL-10, IL-12p70, IL-12p40, IL-16, IL-18, IL-1α, IL-2RA, IL-3, IL-13, IL-15, IL-17, basic fibroblast growth factor (FGF), cutaneous T-cell attracting chemokine (CTACK), eotaxin, granulocyte colony-stimulating factor (G-CSF), granulocyte macrophage colony-stimulating factor (GM–CSF), growth regulated (GRO)-α, hepatocyte growth factor (HGF), interferon (IFN)-γ, IFN-α2, interferon gamma-induced protein (IP)-10, leukemia inhibitory factor (LIF), monocyte chemotactic protein (MCP)-1, MCP-3, macrophage colony-stimulating factor (M-CSF), monokine induced by gamma-Interferon (MIG), macrophage migration inhibitory factor (MIF), macrophage inflammatory protein (MIP)-1α, MIP-1β, nerve growth factor (NGF)-β, platelet derived growth factor (PDGF)-ββ, regulated upon activation normal T cell expressed and presumably secreted (RANTES), stem cell factor (SCF), stem Cell Growth Factor (SCGF)-β, stromal derived factor (SDF)-1α, tumor necrosis factor (TNF)-α, TNF-β, TNF-related apoptosis inducing ligand (TRAIL), and vascular endothelial growth factor (VEGF). The sensitivity of these kits ranged between 0.2 and 45.2 pg/ml for each of the 48 cytokines measured. Data was collected using Bio-Plex Manager software version 6.1, and a 5 PL regression formula was used to calculate sample concentrations from the standard curves. Cytokine levels below the lower limit of detection (LLOD) of the assay were reported as the mid-point between zero and the lowest concentration measured for that given cytokine. To control for inter-plate variability, CVL samples from the same participant were analyzed on separate plates.

### Statistical analyses

Baseline characteristics were summarized using medians with interquartile ranges (IQR) for continuous variables and frequencies and proportions for categorical variables. Proportions were compared using the McNemar test and Bowkers test. The Wilcoxon signed rank test and paired t-tests were used to compare concentrations of isotypes and IgG subclasses for case-matched-control analyses. Multivariable linear mixed models with a random effect of the matched pairs were used to determine associations between cytokines and Ig isotypes and IgG subclasses. The models were adjusted for age, tenofovir use, HSV-2, the number of vaginal sex acts, contraceptives, male condom use and HIV infection status. GI was defined as having at least 5 of 9 elevated cytokines in the 75th upper quartile using previously published scoring criteria^2^. Mann–Whitney *U* test and unpaired *t*-test were performed to compare isotype and IgG subclasses between women with GI versus women without GI within cases and controls. Similar tests were also used for the stratified analyses based on tenofovir use within cases and controls to compare isotype and IgG subclasses (supplementary data). Cytokine concentrations were log transformed to ensure normality. *P*-values were adjusted for multiple comparisons using the Benjamini–Hochberg method. All statistical analysis were performed using GraphPad Prism Version 8.4.3 and SAS version 9.4 (SAS Institute Inc., Cary NC, USA).

## Results

### Study participants

In this case–control sub-study, a total of 132 mucosal specimens of women from CAPRISA 004 (N = 90) and CAPRISA 008 (N = 42) trials were analyzed. Of these, cases represent women (n = 66) at their pre-HIV infection timepoint who subsequently became HIV-infected. Controls represent women (n = 66) who remained HIV-uninfected and were matched to cases by time in study. No significant differences were found between cases and controls with respect to age, education, relationship status, condom use, lifetime number of partners, HSV-2 positivity at time of study enrolment (Table 1). The majority of women (91.7%) reported being in a stable relationship. Age of sexual debut was significantly lower in cases [median 17 years; interquartile range (IQR), 16–18 years] compared to controls and (18; IQR, 16–19; *p* = 0.008). Depo-Provera (DMPA) was the most common contraceptive method used in both groups. Herpes simplex virus-2 (HSV-2) seropositivity was observed in 74% of the cases and 65% of the controls. Active sexually transmitted infections (STIs) such as *Chlamydia, Gonorrhoea* and *Trichomonas* tests were performed in 42 women from CAPRISA 008 only. Of these, six cases and one control had STIs.

**Table 1:** Baseline characteristics of case and controls included in this study.

Demographics	Cases (N = 66)	Controls (N = 66)	*P* value
Age in years, median (IQR)	23 (21–27)	24 (21–28)	0.310
**Highest level of education completed:**			0.446
Less than primary, % (n)	19.7 (13)	7.6 (5)	
Primary Complete, % (n)	34.8 (23)	43.9 (29)	
High school complete, % (n)	42.4 (28)	47 (31)	
Tertiary complete, % (n)	3.0 (2)	1.5 (1)	
**Relationship status:**			0.704
Married, % (n)	4.5 (3)	9.1 (6)	
Stable partner, % (n)	84.8 (56)	84.8 (56)	
Casual partner, % (n)	6.1 (4)	1.5 (1)	
Other, % (n)	4.5 (3)	4.5 (3)	
**Sexual behaviour:**			
Age (years) of sexual debut, median (IQR)	17 (16–18)	18 (16–19)	0.008
Number of lifetime sexual partners, median (IQR)	2 (1.5–4)	2 (1–3)	0.323
Number of vaginal sex acts in the last 30 days Median (IQR)	4 (3–8)	6 (3–10)	0.060
**Contraceptive use:**			*
Depo-Provera, % (n)	71.2 (47)	71.2 (47)	
Oral contraceptive, % (n)	10.6 (7)	13.6 (9)	
Nur-isterate, % (n)	18.2 (12)	7.6 (5)	
Tubal Ligation, % (n)	0	7.6 (5)	
**Condom use:**			0.481
Always % (n)	34.8 (23)	31.8 (21)	
Sometimes % (n)	59.1 (39)	65.2 (43)	
Never % (n)	6.1 (4)	3.0 (2)	
**STI Testing**			
Proportion HSV-2 positive % (n)	75.4 (49)	64.6 (42)	0.26

IQR, interquartile range. HSV-2, Herpes simplex virus-2. A *p*-value of < 0.05 was regarded as statistically significant.

*The Bowker test of symmetry failed as there were no events hence the p-value could not be computed.

### Higher mucosal IgM in cases at pre-HIV infection timepoint

**Figure 1:**
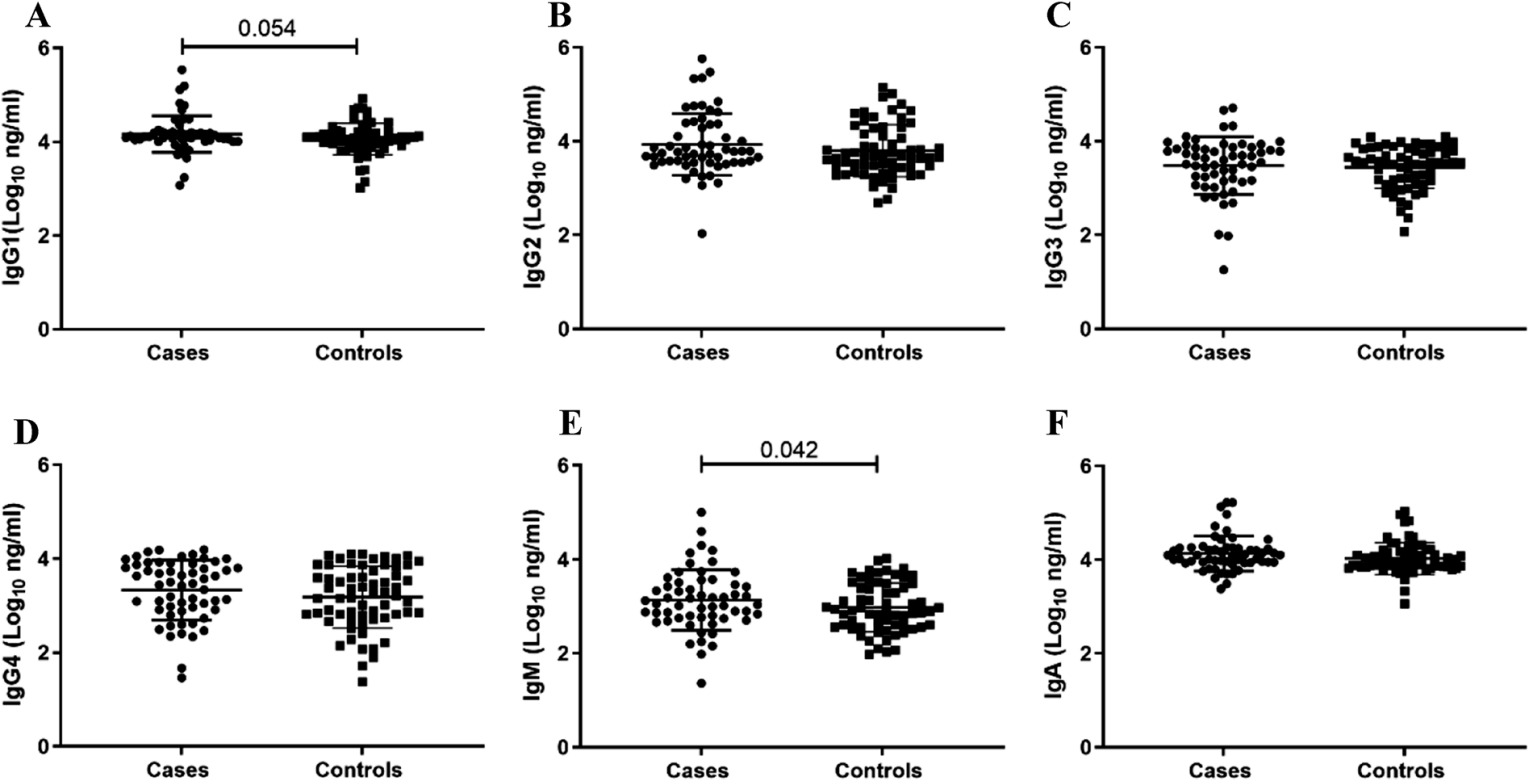
Comparison of mucosal immunoglobulin IgG subclasses and isotypes (Log_10_ ng/ml) between matched controls and cases pre-HIV infection. Each data point represents an individual sample. Cases represent women who subsequently became HIV-infected, and controls represent women who remained HIV-uninfected. The scatter dot plot includes the medians and interquartile ranges. Wilcoxon signed rank test and paired t-tests were used to compare between the case-matched-control groups and *p* < 0.05 were considered statistically significant.

We investigated mucosal IgG subclasses and isotypes in the FGT to determine their differences in the women who become HIV-infected compared women who remained uninfected (Fig. 1). IgM was significantly higher in cases (4.13; IQR, 4.04–4.19) compared to controls (4.06; IQR, 3.90–4.20; *p* = 0.042) (Fig. 1E). IgG1 trended higher in cases (3.05; IQR, 2.76–3.48) than for controls (2.90; IQR, 2.57–3.46; *p* = 0.054) (Fig. 1A). IgG2, IgG3, IgG4 and IgA were similar between cases and controls (Fig. 1B–D and F).

### Mucosal IgG and IgM significantly increases with genital inflammation

**Figure 2:**
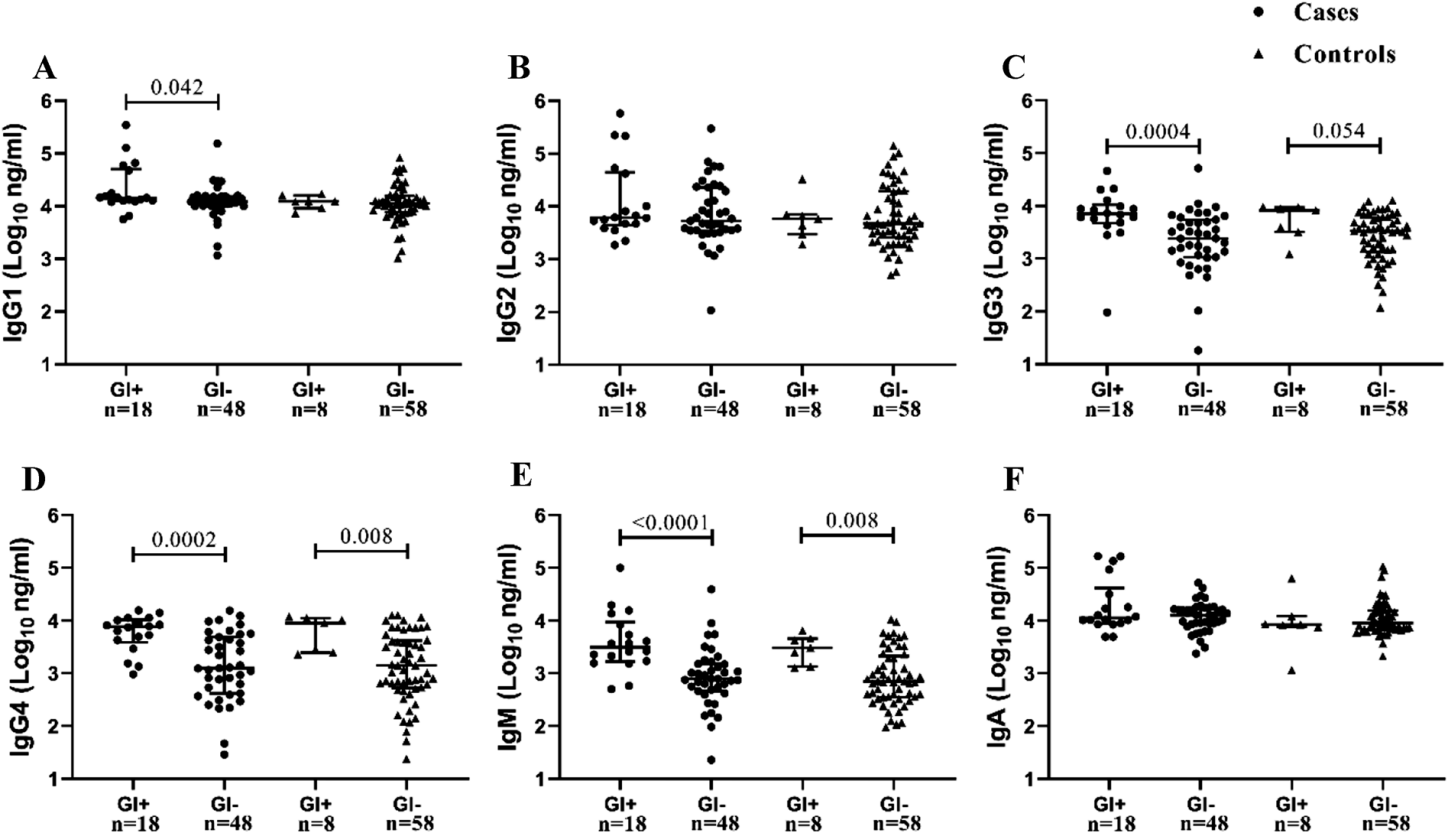
Comparison of mucosal IgG subclasses and isotypes in women stratified for presence of genital inflammation (GI+) or absence of genital inflammation (GI−) within cases [GI+ (n = 18) and GI− (n = 48)] and controls [GI+ (n = 8) and GI− (n = 58)] pre-HIV infection. Each data point represents an individual sample, the line-bars represents medians and interquartile ranges. Mann–Whitney *U* test and unpaired *t*-tests were used to compare between groups and *p* < 0.05 were considered statistically significant. Black circle represents cases and black triangle represents controls.

We next determined the effects of GI on antibodies by further stratifying women according to the presence (GI+) or absence (GI−) of GI irrespective of HIV infection. Among cases, women with GI had significantly higher IgG1 (*p* = 0.042), IgG3 (*p* = 0.0004), IgG4 (*p* = 0.0002) and IgM (*p* < 0.0001) compared to women without GI (Fig. 2A and C–E). We found no significant difference for IgG2 and IgA among GI+ and GI− women (Fig. 2B and F). IgG3, IgG4 and IgM remained significant even after multiple comparisons adjustment. Similarly, in controls, women with GI had significantly higher concentrations of IgG4 (*p* = 0.008), IgM (*p* = 0.008) (Fig. 2D and E) and IgG3 trended higher (*p* = 0.054) (Fig. 2C) compared to women without GI, with IgG4 and IgM remaining significantly higher after multiple comparisons adjustment. This finding highlights the differential profile of antibodies in the presence of inflammation. In order to understand the effect of tenofovir in modifying genital antibodies we further stratified cases and controls by tenofovir use (tenofovir and placebo). Within the cases, and irrespective of the treatment arm, whether women used tenofovir or placebo, concentrations of IgG2 (*p* = 0.019), IgG3 (*p* = 0.002), IgG4 (*p* = 0.0001) and IgM (*p* = 0.004) were significantly higher in women with GI compared to women without GI (Supplementary Fig. S1B–E). Similarly, among the controls, significantly higher IgG3 (*p* = 0.045) and IgM (*p* = 0.008) were observed in women with GI compared to women without GI (Supplementary Fig. S2C and E).

### Genital inflammatory cytokines significantly associated with IgG subclasses, IgM and IgA isotype

**Figure 3:**
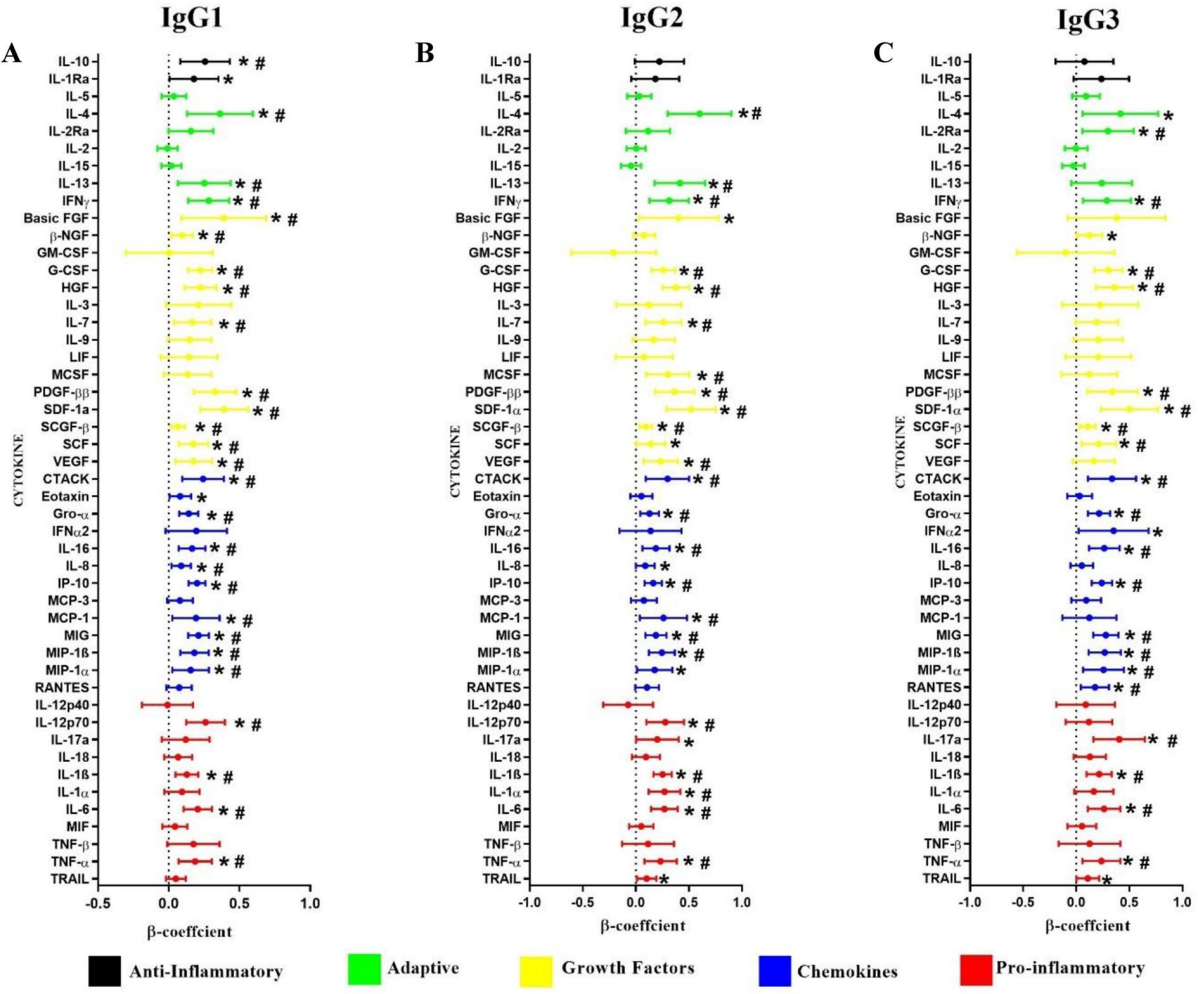

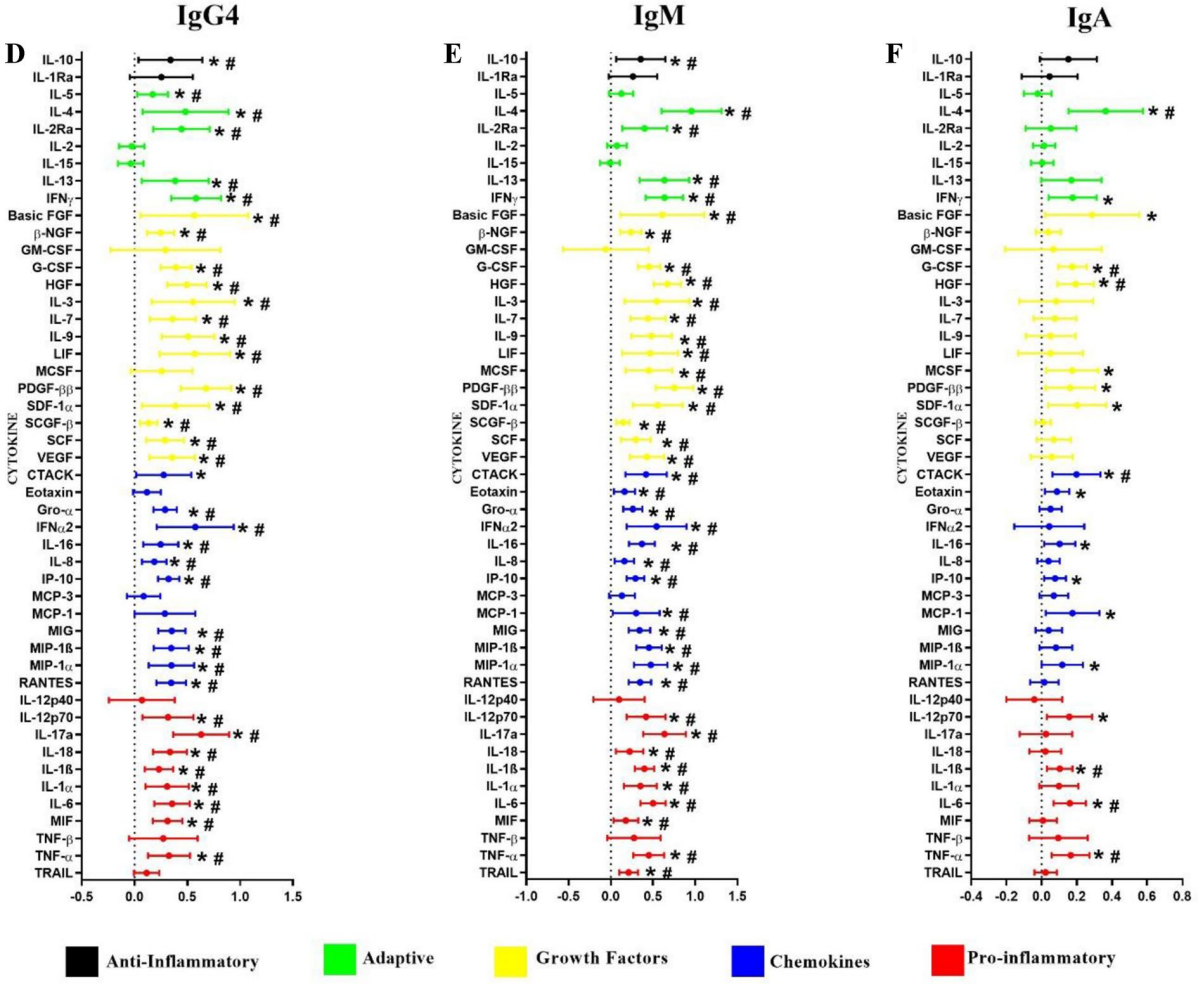
Associations between genital cytokines and mucosal IgG1 (**A**), IgG2 (**B**), IgG3 (**C**), IgG4 (**D**), IgM (**E**) and IgA (**F**). β-coefficients and p-values were determined using multivariable linear mixed models with a random effect of the matched pairs (N = 132). The multivariable models were adjusted for age, HSV-2 status, the number of vaginal sex acts, contraceptive method, condom use, tenofovir use and HIV infection status. β-coefficients are indicated by shaded circles and error bars indicate 95% confidence intervals. *P*-values < 0.05 are represented by *, and those p-values that are significant after multiple comparisons adjustment are represented by #.

Multivariable linear mixed models with a random effect of the matched pairs were performed to determine the associations between the genital cytokines and immunoglobulin isotypes and IgG subclasses. This model was adjusted for age, HSV-2 status, the number of vaginal sex acts, contraceptive method, condom use, tenofovir use and HIV infection status. Most notably, subclass IgG2 and isotype IgM were significantly and positively associated with all the nine cytokines that defined GI (IL-6, IL-8, IP-10, IL-1α, IL-1β, TNF-α, MCP-1, MIP-1α and MIP-1β) (*p* < 0.05), all except MIP-1α remained significant after multiple comparison adjustment (Fig. 3B and E). In addition, IgG1 and IgG3 significantly and positively associated with eight (TNF-α, IL-6, IL-8, IP-10, IL-1β, MCP-1, MIP-1α and MIP-1β) and six (IL-6, IP-10, IL-1β, TNF-α, MIP-1α and MIP-1β) cytokines (*p* < 0.05) respectively (Fig. 3A and C). IgG4 also showed positive and significant associations with all eight cytokines that fits the definition of GI, excluding MCP-1 (*p* < 0.05) and remained significant after multiple comparison adjustment (Fig. 3D). IgA was positively associated with six of nine genital inflammatory cytokines (IL-6, IP-10, IL-1β, TNF-α, MCP-1, MIP-1α) (Fig. 3F) and TNF-α, IL-6 and IL-1β remained significant after multiple comparison adjustment. Besides the nine key pro-inflammatory cytokines, subclasses IgG1-IgG4 and isotype IgM and IgA were significantly and positively associated with several anti-inflammatory, adaptive, growth factors and chemotactic cytokines (*p* < 0.05) (Fig. 3). Together, these data underscore the compartment-specific associations between cytokines and antibody subclasses and isotypes.

## Discussion

The majority of HIV infections in women are acquired during sexual intercourse, making it imperative to understand the immune microenvironment of the FGT for developing strategies to prevent infections. A significant effort has been made to understand if and how antibodies can confer protection in the FGT. Given the significant evidence of GI and increased HIV risk, very little is known about how GI can impact the profile and functions of antibodies in the genital tract. Here, we detected that in the presence of pre-existing GI, mucosal IgG subclasses and isotypes were significantly increased in both women who became HIV-infected and in those who remained uninfected.

In this case–control analyses we found significantly higher IgM in cases compared to controls which may indicate that mucosal IgM can be an early marker of pathogen exposure. Circulating IgM are elicited during the early stage in response to any infection including HIV^29^. Moreover, HIV-specific IgM were functionally effective in neutralizing and reducing HIV infections in cervicovaginal tissue models^30^. Natural IgM can limit HIV infection by modulating inflammation and T cell activation^31,32^. In addition, IgM can directly bind to CD4+T cells and chemokine receptors CCR5, hindering HIV entry^33^. Together, these data highlight the varied functional capability of pentameric IgM to effectively crosslink and capture virus thereby reducing HIV infection. Upon class switching from IgM, IgG1 predominates during acute and chronic HIV infection^11^. We also found higher IgG1 in cases compared to controls. HIV-specific IgG1 have been associated with viral control and slower progression to HIV infection^34,35^. These data suggest that in the cases, at the pre-HIV infection stage, sexual exposure to HIV or other pathogens may have elicited increased mucosal IgG1 and IgM titres. However, whether these antibodies are functionally competent and HIV-specific IgG or IgM, remains undefined.

To determine genital antibody profiles in the presence of GI, women were stratified for the presence or absence of GI. IgM, IgG1, IgG3 and IgG4 were significantly higher in women with GI compared to women without GI. We previously reported higher titer of HIV-specific IgG and IgA in the genital tracts of women with breakthrough HIV infections who used tenofovir gel from the CAPRISA 004 trial^36^. However, in this study we found that irrespective of tenofovir use there was significantly increased IgG2, IgG3, IgM and IgG4 in the presence of GI. Together these data infer that tenofovir gel use was less likely than GI to modify genital antibodies. However, we cannot over interpret these data and the limited sample size in each sub-category precludes accurate conclusions. Both in vivo and in vitro studies suggested that IgM increases as a compensatory drive to resolve inflammation^32,37^. Therefore, increased mucosal IgM among women with GI may be in response to the inflammation. Studies have demonstrated cytokine-induced enhancement of IgG subclasses and isotypes^38,39^ explaining in part the increased antibodies with GI. Alternatively, compromised barrier integrity may also account for the increased antibody transudate in the presence of GI. Inflammation is strongly associated with the glycosylation of the fragment crystallizable (Fc) region of IgG subclasses particularly^40^. This reduces antibody affinities for Fcγ receptors decreasing functional activities^41,42^. Besides glycosylation, increased proteases have been associated with elevated inflammatory cytokines in the FGT^7^ which can lead to non-specific proteolytic antibody degradation compromising the function of the antibodies^24^. Therefore, the impact of GI on antibody functions in the genital tract need to be investigated in future studies. Nevertheless, the findings suggest that inflammatory environment in the FGT may be responsible for maintaining specific antibody responses, but this needs further investigation in large cohort studies.

Cytokines have been shown to influence Ig class switching and subclass synthesis^39,43^. In this study we demonstrated a link between genital inflammatory cytokines and mucosal antibodies. We found that prior to HIV infection, mucosal IgG subclasses and isotypes were positively and significantly associated with several inflammatory cytokines: TNF-α, IL-1α, IL-1β, IL-10 and IL-6; adaptive cytokines: IL-13, IL-4 and IFN-γ and chemotactic and growth factors: MIP-1α, MIP-1β, MCP-1, IL-8, CTACK and MIG after adjusting for potential confounders. Positive associations were observed between IL-13 and IgG4 and IgM. These data corroborate previous findings of IL-13 induced synthesis of IgM and IgG4 in human B cells in the presence of activated T cells^44^. Furthermore, IL-6 along with IFN-γ can inhibit IgG1 and enhance IgG2 production in stimulated PBMCs demonstrating the differential regulation of cytokines on human IgG subclass production^39^. The significantly direct association between IL-7 and IgG4 is also supported by a study showing IL-7 driven class switching to IgG4^45^. Collectively, these preliminary findings suggest that the local isotypes and IgG subclasses may be influenced, in part, by pre-existing GI.

The strength of the study is the evaluation of rare pre-HIV infection mucosal samples from high-risk South African women in two clinical trials investigating mucosal Ig isotype/subclasses in relation to cytokines. Mucosal antibody profiling may be an additional predictor of pre-existing GI. However, these studies would indeed require large cohorts evaluating both mucosal cytokines and antibodies longitudinally and in parallel to establish their validity as an additional surrogate for GI. Understanding the association between GI and antibodies can provide further insight for developing effective HIV prevention strategies for at risk populations with underlying susceptibilities to GI. This study however has several limitations. We did not measure pathogen-specific antibodies, an important surrogate indicator of bacterial and viral pathogen exposure. All the participants were on some form of hormonal contraceptives, a mandatory inclusion criterion for trial participation. Therefore, we could not account for phase of the menstrual cycle, which is known to affect inflammatory cytokines and antibodies^46,47^. A previous study of women on DMPA showed significantly increased IgG1, IgG2 and IgA and IL-1α, IL-1β, IL-2, MIP-1β, IP-10, IL-8 and TGF-β cytokines in the FGT^48^. These data affirm the local impact of exogenous hormones on immune responses in the FGT. In this study we adjusted for hormonal contraceptive use as one of the potential confounders in the linear mixed model. However, further studies are needed to understand the impact of endogenous and exogenous hormones on GI and mucosal antibodies.

Another caveat to the present study is the lack of data for STIs and BV in the CAPRISA 004 trial which precluded the ability to establish significant associations of GI with STIs and BV. Previous studies have shown that both STIs and BV can induce inflammation in the genital tract impacting the mucosal immune responses^49,50^. Inflammatory cytokines in the FGT were similar in women with symptomatic and asymptomatic STIs^51^. In addition, women with asymptomatic STIs had significantly elevated cytokines compared to women with no STIs or BV^51^. Toll-like receptors (TLRs) recognize pathogens including STIs inducing specific T-cell and antibody response^52,53^. In addition, TLRs recognize pathogen associated molecular patterns (PAMPs) of bacterial STIs inducing NF-κB driven immune response resulting in inflammatory cytokine production ^54,55^. Therefore, to exclude the bias of GI due to STIs and BV, these two factors should be adjusted for as confounders. Future studies would, therefore, require large sample sizes to account for the selection bias and confounding of GI secondary to STIs and BV. Despite these shortcomings, our data provides an enhanced dissection of how GI can affect the quantities of mucosal antibodies and, potentially impact their functions through the predominance of certain isotypes or subclasses over the other. Our findings suggest that GI can shape or reconfigure the mucosal Ig subclass and isotype signature. However, further investigation is required to verify a plausible link between the local inflammatory milieu and the effect on genital antibodies and their functions. These data may be important for future HIV vaccine efficacy studies.

## Supplementary Information

Supplementary Information.
