# Supplementary material for: Higher mucosal antibody concentrations in women with genital tract inflammation
Source: Sci Rep. 2021 Dec 6;11:23514. doi: 10.1038/s41598-021-02954-0 (PMC8648917; doi:10.1038/s41598-021-02954-0)
Supplement: Supplementary file 1 — Supplementary Information. [file 41598_2021_2954_MOESM1_ESM.docx]

**Supplementary Figure 1**


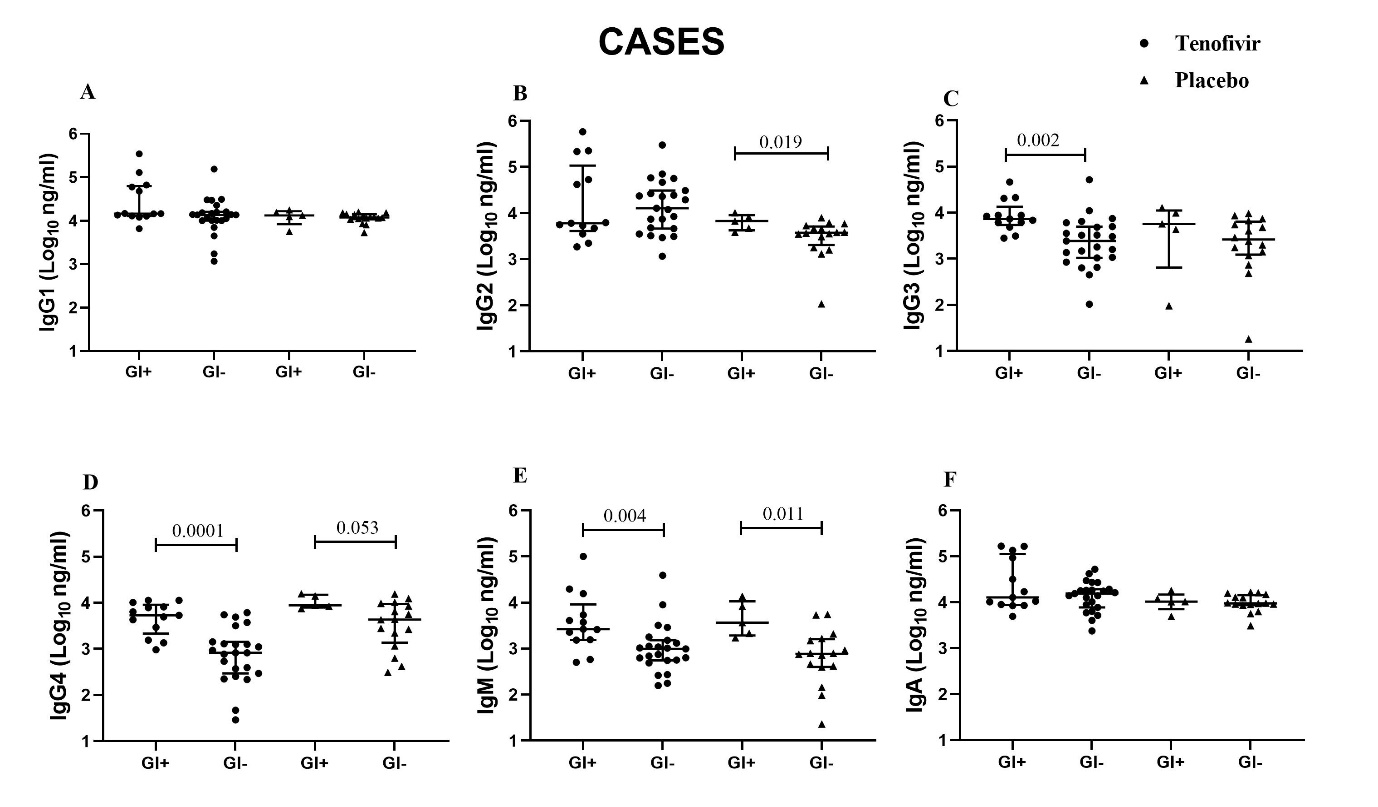


**Supplementary Figure 1.** Comparison of mucosal IgG subclasses and isotypes in cases stratified for presence of genital inflammation (GI+) or absence of genital inflammation (GI-) within tenofovir and placebo groups. Each data point represents an individual sample, the line-bars represents medians and interquartile ranges. Mann-Whitney U test and unpaired t-tests were used to compare between groups and p<0.05 were considered statistically significant. ● represent tenofovir use and ▲ represent placebo.

**Supplementary Figure 2**


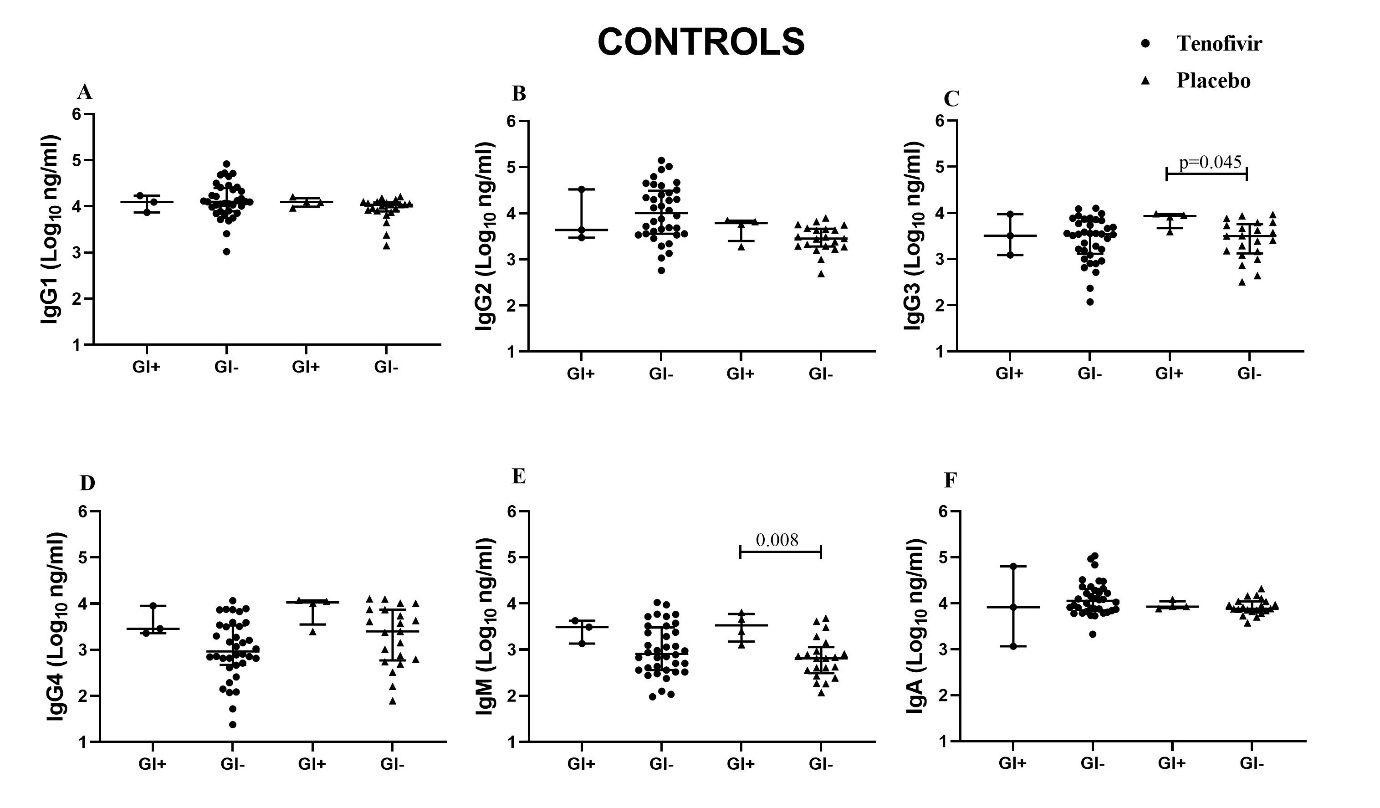


**Supplementary Figure 2.** Comparison of mucosal IgG subclasses and isotypes in controls stratified for presence of genital inflammation (GI+) or absence of genital inflammation (GI-) within tenofovir and placebo groups. Each data point represents an individual sample, the line-bars represents medians and interquartile ranges. Mann-Whitney U test and unpaired t-tests were used to compare between groups and p<0.05 were considered statistically significant. ● represent tenofovir use and ▲ represent placebo.
